# Evaluation of occupational fatigue among Chinese nursing managers: a cross-sectional online study

**DOI:** 10.3389/fpubh.2026.1752771

**Published:** 2026-01-30

**Authors:** Jiabing Wu, Yonghong Wang, Qiuyang He, Xueying Xun, Guoyu Wang

**Affiliations:** 1 Department of Obstetric Nursing, West China Second University Hospital, Sichuan University, Chengdu, China; 2 Key Laboratory of Birth Defects and Related Diseases of Women and Children (Sichuan University), Ministry of Education, Chengdu, China

**Keywords:** Chinese, cross-sectional online study, influencing factors, nursing managers, occupational fatigue

## Abstract

**Introduction:**

To evaluate the levels of occupational fatigue and work stress experienced by nursing managers in western China and to identify the factors influencing occupational fatigue.

**Methods:**

This study recruited 248 nursing managers from 186 hospitals across 28 Chinese provinces, of which 91.1% are located in western China and 78.2% are tertiary hospitals. The nursing managers included in this study were actively engaged in their managerial roles throughout the data collection period, discharging associated administrative and clinical responsibilities.

**Results:**

Western Chinese nursing managers reported mid-low to mid-high levels of occupational chronic fatigue, mid-high to high levels of acute fatigue, and mid-low to mid-high levels of inter-shift recovery. Effort and overcommitment were significantly associated with all levels of fatigue. Chronic fatigue was associated with hospital grade, while acute fatigue was associated with hospital grade, position title, and working hours per week. Inter-shift recovery was linked to weekly working hours.

**Conclusion:**

Effort and overcommitment were significantly associated with occupational fatigue levels among western Chinese nursing managers. Additionally, fatigue severity correlated with hospital grade, weekly working hours, and position title. Policy guidance, organization support, and work competency training may be beneficial.

## 1 Introduction

Fatigue is a widespread issue, impacting approximately 16.4% of the global population, with general fatigue affecting 20.4% of adults (1). Various factors, including gender, age, occupation, economic and cultural differences, and geographic location, contribute to fatigue (1, 2). Specifically, regarding occupational factors, professions such as nurses, pilots, and healthcare workers exhibit a notably higher prevalence of fatigue compared to the general adult population (1). Among healthcare professionals, occupational fatigue is characterized by cumulative exhaustion and diminished energetic resources that arise when physical and mental demands chronically exceed recovery opportunities (3). Nurses, owing to extended working hours, night-shift rotations, work-related stress, physical workload, and chronic sleep deficit (4, 5), are considered the healthcare subgroup particularly susceptible to occupational fatigue (6, 7).

Nursing managers constitute a unique, under-researched subset of the workforce. According to the American Nurses Association Scope and Standards for Nurse Leaders, they are accountable for quality governance, strategic workforce development, human-resource allocation and maintenance of a healthy practice environment (8). Although few studies have investigated occupational fatigue among nursing managers compared to the extensive research conducted on clinical nurses, the available findings suggest that nursing managers experience significant levels of occupational fatigue (9, 10). The global shortage of nursing personnel has magnified these pressures in both the workforce and nursing practice (11, 12), with a projected deficit of 13 million by 2030 (13). In China, an ageing population, tiered referral reforms and aggressive hospital expansion have escalated nurse demand, forcing managers to reconcile stringent quality indicators with chronically understaffed units (14, 15). Persistent fatigue at the managerial level undermines decision accuracy, erodes job satisfaction and predicts turnover intention, thereby threatening care continuity (16, 17). Nevertheless, no multi-center study has simultaneously quantified fatigue intensity and associated work stressors among Chinese nursing managers. Addressing this evidence gap is essential for designing targeted recovery interventions and sustainable leadership policies. We conducted an exploratory, cross-sectional survey of Chinese nursing managers, primarily located in western China.

## 2 Background

### 2.1 Conception of fatigue

Chronic fatigue denotes a persistent, sustained state integrating affective, cognitive and somatic components, whereas acute fatigue reflects transient incapacity that manifests immediately after task cessation (18–20). Inter-shift recovery refers to the degree to which acute fatigue dissipates between duty cycles, the persistent accumulation of fatigue can trigger a vicious cycle of chronic fatigue (18–20). Insufficient recovery compromises subsequent performance and elevates error probability (20).

### 2.2 Theoretical frameworks

The Occupational Fatigue Exhaustion/Recovery (OFER) model, developed and validated by Winwood et al. (18, 19), operationalizes these dynamics through three correlated sub-scales: chronic fatigue, acute fatigue and inter-shift recovery. The instrument was originally normed on hospital nurses but has been cross-validated in allied health professionals, including nursing managers (20, 21).

Complementarily, Effort–Reward Imbalance (ERI) model by Siegrist reveals that sustained discrepancy between high extrinsic effort and low extrinsic reward elicits sustained stress arousal, thereby precipitating adverse health outcomes (22, 23). Meta-analytic evidence connects ERI with depression, anxiety, metabolic dysregulation and, critically, occupational fatigue (23, 24).

ERI theory locates the psychosocial source of work-related ill health—effort-reward imbalance—whereas the OFER quantifies its expression, occupational fatigue. Their integration yields a multilevel risk architecture that identifies vulnerable employees and informs interventions.

### 2.3 Application to western Chinese nursing managers

China’s healthcare context is likely to amplify the fatigue trajectory outlined by the OFER and ERI models. Hospitals are simultaneously experiencing a rapid increase in patient acuity driven by an ageing population. Culturally, Chinese nursing managers tend to score high on collectivism and prioritize individual sacrifice for the sake of group harmony. This norm implicitly endorses the practice: many western Chinese nursing managers voluntarily remain on site after formal handover to ensure ward stability, effectively compressing inter-shift recovery time.

## 3 Materials and methods

### 3.1 Research design

In this cross-sectional study, data collection was facilitated through an online survey. A non-probability convenience sampling method was employed, resulting in a sample composition of 24.6% deputy nurse managers, 57.3% nurse managers, 10.5% departmental nurse managers, and 7.6% directors of nursing departments. At the inception of the study, formal consultation was sought from the Chinese Nursing Association (CNA) to delineate the objectives and methodology. This association holds the preeminent authority concerning the representation of the nursing profession within China, with the majority of its members occupying roles such as directors of nursing departments across various regions of the country. Prior to data collection, the OFER and ERI scales were adapted for use in a managerial population. Content validity was assessed through an expert review by five specialists in occupational health and work-related stress, who rated the relevance and clarity of each item on a 4-point scale. The scale-level content validity index (S-CVI/Ave) reached 0.933 for the OFER and 0.913 for the ERI, both exceeding the 0.90 criterion for excellent content validity. Because the research team was based in western China, recruitment prioritized local individual CNA members, resulting in a sample dominated by nursing managers from that region. A total of 266 questionnaires were distributed through the CNA network. All participants held managerial positions in the nursing field. In accordance with ethical standards, all participants received an informational sheet prior to enrollment detailing the study’s purpose, anticipated duration, participant rights, and privacy protection policies.

### 3.2 Participants

Inclusion criteria were as follows: (1) Nurses in managerial positions; (2) Nurses with no language, reading, or other cognitive impairments were observed. Exclusion criteria were: (1) Nursing managers who did not formally hold a position of responsibility; (2) Nursing managers in further studies and visiting scholars; (3) Retired nursing managers (nursing managers who had retired and stepped down from their positions at the time of the survey); (4) Nursing managers on leave of absence (e.g., maternity, sick, paid/unpaid leave of absence) during the data collection period; (5) Those participating in other fatigue intervention studies. The sample size for this study was determined using the formula N ≥ 8(m) + 50 (25), where “m” denoted the number of independent variables (m = 15), and “N” represented the sample size. Taking into account the possibility of 20% missing data, the expected minimum sample size was 213. A total of 266 questionnaires were distributed, and 254 were returned. We excluded any response that met at least one of the following pre-specified exclusion criteria: (1) ≥ 20% missing data, (2) completion time < 3 min. Ultimately, 248 participants were included in the analysis (*N* = 248).

### 3.3 Data collection procedures

Data were collected from January 17 to March 16, 2023. The survey was conducted using an easily accessible and user-friendly online platform, Survey Star.

### 3.4 Research instruments

The survey instrument comprised two sections: one addressing the general information pertinent to the study and the other focusing on occupational fatigue and stress queries. The general information section was structured to gather crucial demographic and occupational data, encompassing variables such as gender (men and women), age (20–30 years, 31–40 years, 41–50 years and >50 years), marital status (unmarried, married and divorced or widowed), educational attainment (three-year college, undergraduate, and master’s degree or above), hospital location (eastern part, central part, and western part), hospital grade (primary hospital, secondary hospital, and tertiary hospital), years of experience (6–10 years, 11–20 years, and >20 years), position title (deputy nurse managers, nurse managers, departmental nurse managers, and directors of nursing departments), professional title (nurse practitioner, nurse-in-charge, associate professor of nursing and professor of nursing), department (Supplementary Table S1), working hours per week (≤40 h, 41-45 h, 46-50 h, and >50 h), and the frequency of night shifts per month (0 shift, 1–4 shifts, and ≥5 shifts).

The assessment of occupational fatigue was conducted utilizing the Occupational Fatigue Exhaustion/Recovery Scale (OFER) (18–20). Chronic fatigue is defined as a complex psychological, physiological, and emotional state that encompasses multiple components, including a depressive mood (18–20). Acute fatigue is a relative state of “incapacity” resulting directly from fatigue caused by previous activities, which is most evident after work (18–20). Inter-shift recovery refers to the extent to which perceived acute work-related fatigue dissipates between consecutive work shifts. Insufficient inter-shift recovery may lead to persistent fatigue at the start of the next shift, thereby affecting work efficiency and quality (18–20). The OFER consists of three dimensions, each comprising five items. Using a 7-point Likert scale with scores ranging from 0 to 6, corresponding to “strongly disagree” responses to “strongly agree,” and items 9, 10, 11, 13, and 15 were reverse-scored (18–20). Each dimension was assessed by calculating a score derived from dividing the total number of items by 30 and multiplying by 100. The resulting scores were categorized as follows: 0–25 indicated low levels, 26–50 indicated mid-low levels, 51–75 indicated mid-high levels, and 76–100 indicated high levels of occupational fatigue (20, 21). A higher score corresponded to an elevated level of chronic fatigue, acute fatigue, or inter-shift recovery. Fang et al. evaluated the Chinese version of the OFER scale among nurses in mainland China and verified its robust psychometric properties (20). Their study reported Cronbach’s alpha values of 0.83, 0.85, and 0.86 for chronic fatigue, acute fatigue, and inter-shift recovery subscales, respectively (20). In the present study, we utilized the Chinese version of the OFER scale, and the Cronbach’s alpha values were determined to be 0.89, 0.87, and 0.78, respectively.

The Effort-Reward Imbalance (ERI) scale (22, 23, 26), is extensively utilized to assess perceived work-related stress, particularly among nurses. This scale comprises 23 items distributed across three dimensions: effort, reward, and overcommitment. Responses to the items in the effort and reward domains are rated on a scale from 1 to 5, where a score of 1 signifies no stressful experience, and a score of 5 signifies a very high stress level. The overcommitment domain items are rated on a scale from 1 to 4, with a score of 4 indicating complete agreement with the statement. The total scores range from 6 to 30 for the effort domain, 11 to 55 for the reward domain, and 6 to 24 for the overcommitment domain. Items 7 through 17 are reverse-scored. Notably, work-related stress is a pivotal component of the ERI model, which is frequently employed to evaluate the impact of work-related stress on individuals’ physical and mental health by examining the imbalance between input and output (22, 23, 26). Work-related stress can be quantified using the Effort-Reward Ratio (ERR) and overcommitment. Effort-Reward Ratio (ERR) is calculated by dividing the effort score by the reward score, which is then multiplied by a correction factor of 0.5454 to account for the number of items assessed (26). A value of 0 suggests a positive working environment characterized by a manageable workload and greater profits, while a value exceeding 1.0 signifies a substantial discrepancy between effort and performance (26). The Chinese adaptation of the ERI scale has exhibited strong psychometric properties, as evidenced in prior research (26). In the present study, Cronbach’s alpha coefficients for the effort, reward, and overcommitment subscales were reported as 0.90, 0.89, and 0.77, respectively.

### 3.5 Data analyses

The data analysis was conducted utilizing SPSS software, version 27.0. The demographic and occupational variables were categorical data. Some demographic variables (marital status, education, department, and night shifts per month) were dichotomized at the median value of the sample; no statistically significant differences were observed before or after dichotomization (all *p* > 0.05). Data from the OFER scale and the ERI scale exhibited non-normal distribution. Based on predefined criteria (detailed as OFER scale brief) (20, 21), we categorized OFER scores of chronic fatigue, acute fatigue, and inter-shift recovery into four groups (e.g., chronic fatigue levels for low, mid-low, mid-high, and high). Comparative analysis between demographic or occupational variables and the OFER scale was performed using the Chi-Square. Categorical associations were first examined with Pearson’s χ^2^ test. When more than 20% of cells had expected counts below 5, the Fisher–Freeman–Halton (FFH) exact test was used. If the complete enumeration required for the FFH test exceeded the default 5-min time limit in SPSS, we replaced it with the Monte Carlo exact method based on 10,000 random tables; 99% confidence intervals for the *p*-values are reported. We assessed the correlation between OFER and ERI via Spearman correlation analysis. After checking the proportional-odds assumption, we fitted the appropriate ordinal or multiple logistic regression model and included variables significant at *p* < 0.05 to estimate their associations with occupational fatigue.

### 3.6 Ethical considerations

All procedures performed in studies involving human participants were in accordance with the ethical standards of the Ethics Committee of West China Second Hospital of Sichuan University (Ethics No.: 2020114) and with the 1964 Helsinki declaration and its later amendments or comparable ethical standards. Informed consent was obtained from all individual participants included in the study.

## 4 Results

The sample predominantly comprised married female nurse managers who were middle-aged and held a bachelor’s degree. They possessed over ten years of work experience and held the professional title of nurse-in-charge. The participants were primarily employed in tertiary hospitals located in the western region of China. They typically worked 41–45 h per week and infrequently undertook night shifts (Supplementary Table S1).

### 4.1 The levels of occupational fatigue and work-related stress

In our study, the scores on the three dimensions of the OFER scale (chronic fatigue, acute fatigue, and inter-shift recovery) were found to be non-normally distributed. We summarized them using frequencies and percentages (Supplementary Table S2). Similarly, the three dimensions of the ERI scale (Effort, Reward, and Overcommitment) also exhibited non-normal distributions. These dimensions were characterized using medians and quartiles (Supplementary Table S3). As illustrated, most nursing managers reported experiencing mid-low to mid-high levels of chronic fatigue, mid-high to high levels of acute fatigue, and mid-low to mid-high levels of inter-shift recovery. Concurrently, the median scores for effort and overcommitment were lower than those for the reward score. Furthermore, 64.92% of the ERRs were lower than value 1.

### 4.2 Comparison of different characteristics across various chronic/acute fatigue and inter-shift recovery levels

Table 1 presents the distinct characteristics of nursing managers categorized by varying levels of chronic fatigue, acute fatigue, and inter-shift recovery. Significant differences were identified in hospital grade across chronic fatigue levels (*p* < 0.05). Furthermore, significant differences were noted in hospital grade, professional title, position title, and weekly working hours across varying levels of acute fatigue (*p* < 0.05). Similarly, significant differences were observed in hospital location and weekly working hours among different levels of inter-shift recovery (*p* < 0.05).

**Table 1:** Comparison of different characteristics of nursing managers across various chronic/acute fatigue and inter-shift recovery levels (N = 248).

Categories	Degree of chronic fatigue, *N* (%)				*χ^2^/χ^2^*FH	*p*	Degree of acute fatigue, *N* (%)				*χ^2^/χ^2^*FH	*p*	Degree of inter-shift recovery, *N* (%)				*χ^2^/χ^2^*FH	*p*
	Low	Mid-low	Mid-high	High			Low	Mid-low	Mid-high	High			Low	Mid-low	Mid-high	High		
Gender																		
Male	1	2	1	4	3.13 ^b^	0.38	0	1	5	2	1.95 ^b^	0.56	0	5	3	0	0.80 ^b^	0.66
	(0.40%)	(0.81%)	(0.40%)	(1.61%)			(0.00%)	(0.40%)	(2.02%)	(0.81%)			(0.00%)	(2.02%)	(1.21%)	(0.00%)		
Female	52	71	66	51			25	62	86	67			15	102	80	43		
	(20.97%)	(28.63%)	(26.61%)	(20.56%)			(10.08%)	(25.00%)	(34.68%)	(27.02%)			(6.05%)	(41.13%)	(32.26%)	(17.34%)		
Age																		
20–30 years	1	1	4	5	8.13 ^b^	0.50	1	1	4	5	10.04 ^b^	0.30	2	5	4	0	9.25^b^	0.35
	(0.40%)	(0.40%)	(1.61%)	(2.02%)			(0.40%)	(0.40%)	(1.61%)	(2.02%)			(0.81%)	(2.02%)	(1.61%)	(0.00%)		
31–40 years	27	36	38	31			15	30	47	40			9	57	44	22		
	(10.89%)	(14.52%)	(15.32%)	(12.50%)			(6.05%)	(12.10%)	(18.95%)	(16.13%)			(3.63%)	(22.98%)	(17.74%)	(8.87%)		
41–50 years	23	32	23	18			7	29	36	24			4	43	32	17		
	(11.29%)	(14.92%)	(16.94%)	(14.52%)			(2.82%)	(11.69%)	(14.52%)	(9.68%)			(1.61%)	(17.34%)	(12.90%)	(6.85%)		
>50 years	2	4	2	1			2	2	4	0			0	2	3	4		
	(0.81%)	(1.61%)	(0.81%)	(0.40%)			(0.81%)	(0.81%)	(1.61%)	(0.00%)			(0.00%)	(0.81%)	(1.21%)	(1.61%)		
Marital status																		
Married	51	72	63	48	77.18 ^b^	0.05	24	61	86	63	1.83 ^b^	0.61	13	100	81	40	4.10 ^b^	0.21
	(20.56%)	(29.03%)	(25.40%)	(19.35%)			(9.68%)	(24.60%)	(34.68%)	(25.40%)			(5.24%)	(40.32%)	(32.66%)	(16.13%)		
Unmarried	2	1	4	7			1	2	5	6			2	7	2	3		
	(0.81%)	(0.40%)	(1.61%)	(2.82%)			(0.40%)	(0.81%)	(2.02%)	(2.42%)			(0.81%)	(2.82%)	(0.81%)	(1.21%)		
Education																		
Undergraduate	46	59	57	46	0.91^a^	0.82	23	53	74	58	1.66 ^a^	0.65	12	88	68	40	3.27 ^a^	0.35
	(18.55%)	(23.79%)	(22.98%)	(18.55%)			(9.27%)	(21.37%)	(29.84%)	(23.39%)			(4.84%)	(35.48%)	(27.42%)	(16.13%)		
Non-undergraduate	7	14	10	9			2	10	17	11			3	19	15	3		
	(2.82%)	(5.65%)	(4.03%)	(3.63%)			(0.81%)	(4.03%)	(6.85%)	(4.44%)			(1.21%)	(7.66%)	(6.05%)	(1.21%)		
Hospital location																		
Western part	43	68	64	51	9.97 ^c^	0.09	21	55	86	64	5.75 ^c^	0.40	14	100	78	34	11.48 ^c^	0.04
	(17.34%)	(27.42%)	(25.81%)	(20.56%)			(8.47%)	(22.18%)	(34.68%)	(25.81%)			(5.65%)	(40.32%)	(31.45%)	(13.71%)		
Eastern part	5	1	2	3			2	3	3	3			1	5	1	4		
	(2.02%)	(0.40%)	(0.81%)	(1.21%)			(0.81%)	(1.21%)	(1.21%)	(1.21%)			(0.40%)	(2.02%)	(0.40%)	(1.61%)		
Central part	5	4	1	1			2	5	2	2			0	2	4	5		
	(2.02%)	(1.61%)	(0.40%)	(0.40%)			(0.81%)	(2.02%)	(0.81%)	(0.81%)			(0.00%)	(0.81%)	(1.61%)	(2.02%)		
Hospital grade																		
Tertiary	43	64	52	35	12.51 ^b^	0.03	18	53	78	45	12.99 ^b^	0.03	8	80	69	37	11.02 ^b^	0.06
	(17.34%)	(25.81%)	(20.97%)	(14.11%)			(7.26%)	(21.37%)	(31.45%)	(18.15%)			(3.23%)	(32.26%)	(27.82%)	(14.92%)		
Secondary	8	9	12	16			7	8	10	20			7	23	10	5		
	(3.23%)	(3.63%)	(4.84%)	(6.45%)			(2.82%)	(3.23%)	(4.03%)	(8.06%)			(2.82%)	(9.27%)	(4.03%)	(2.02%)		
Primary	2	0	3	4			0	2	3	4			0	4	4	1		
	(0.81%)	(0.00%)	(1.21%)	(1.61%)			(0.00%)	(0.81%)	(1.21%)	(1.61%)			(0.00%)	(1.61%)	(1.61%)	(0.40%)		
Years of experience																		
6–10 years	5	6	10	11			2	4	10	16			4	19	7	2		
	(2.02%)	(2.42%)	(4.03%)	(4.44%)			(0.81%)	(1.61%)	(4.03%)	(6.45%)			(1.61%)	(7.66%)	(2.82%)	(0.81%)		
11–20 years	23	35	29	23	4.96 ^a^	0.55	144	25	43	28	12.78 ^a^	0.05	6	43	41	2	9.42 ^a^	0.15
	(9.27%)	(14.11%)	(11.69%)	(9.27%)			(5.65%)	(10.08%)	(17.34%)	(11.29%)			(2.42%)	(17.34%)	(16.53%)	(8.06%)		
>20 years	25	32	28	21			9	34	38	25			5	45	35	21		
	(10.08%)	(12.90%)	(11.29%)	(8.47%)			(3.63%)	(13.71%)	(15.32%)	(10.08%)			(2.02%)	(18.15%)	(14.11%)	(8.47%)		
Position title																		
Deputy nurse managers	14	15	17	15	9.28 ^a^	0.41	6	9	26	20	23.03 ^a^	<0.00	4	31	18	8	11.49 ^c^	0.22
	(5.65%)	(6.05%)	(6.85%)	(5.65%)			(2.42%)	(3.63%)	(10.48%)	(8.06%)			(1.61%)	(12.50%)	(7.26%)	(3.23%)		
Nurse managers	29	43	38	32			12	43	52	35			9	56	52	25		
	(11.69%)	(17.34%)	(15.32%)	(12.90%)			(2.82%)	(4.44%)	(5.24%)	(5.65%)			(3.63%)	(22.58%)	(20.97%)	(10.08%)		
Departmental nurse managers	2	10	8	6			1	4	10	11			2	14	8	2		
	(0.81%)	(4.03%)	(3.23%)	(2.42%)			(0.40%)	(1.61%)	(4.03%)	(4.44%)			(0.81%)	(5.65%)	(3.23%)	(0.81%)		
Directors of nursing departments	8	5	4	2			6	7	3	3			0	6	5	8		
	(3.23%)	(2.02%)	(1.61%)	(0.81%)			(2.42%)	(2.82%)	(1.21%)	(1.21%)			(0.00%)	(2.42%)	(2.02%)	(3.23%)		
Professional title																		
Nurse practitioner	1	2	3	8	14.11 ^c^	0.09	0	2	4	8	22.02 ^c^	<0.00	2	9	2	1	14.19 ^c^	0.08
	(0.40%)	(0.81%)	(1.21%)	(3.23%)			(0.00%)	(0.81%)	(1.61%)	(3.23%)			(0.81%)	(3.63%)	(0.81%)	(0.40%)		
Nurse-in-charge	27	39	41	32			15	25	53	46			12	62	44	21		
	(10.89%)	(15.73%)	(16.53%)	(12.90%)			(6.05%)	(10.08%)	(21.37%)	(18.55%)			(4.84%)	(25.00%)	(17.74%)	(8.47%)		
Associate professor of nursing	21	28	22	14			8	32	31	14			1	32	33	19		
	(8.47%)	(11.29%)	(8.87%)	(5.65%)			(3.23%)	(12.90%)	(12.50%)	(5.65%)			(0.40%)	(12.90%)	(13.31%)	(7.66%)		
Professor of nursing	4	4	1	1			2	4	3	1			0	4	4	2		
	(1.61%)	(1.61%)	(0.40%)	(0.40%)			(0.81%)	(1.61%)	(1.21%)	(0.40%)			(0.00%)	(1.61%)	(1.61%)	(0.81%)		
Department																		
Clinical departments	45	65	60	49	0.77 ^a^	0.86	19	54	82	64	5.69 ^a^	0.13	14	95	76	34	4.8 ^a^	0.19
	(18.15%)	(26.21%)	(24.19%)	(19.76%)			(7.66%)	(21.77%)	(33.06%)	(25.81%)			(5.65%)	(38.31%)	(30.65%)	(13.71%)		
Non-clinical departments	8	8	7	6			6	9	9	5			1	12	7	9		
	(3.23%)	(3.23%)	(2.82%)	(2.42%)			(2.42%)	(3.63%)	(3.63%)	(2.02%)			(0.40%)	(4.84%)	(2.82%)	(3.63%)		
Working hours per week																		
≤40 h	8	6	7	4	11.88 ^a^	0.22	4	10	10	1	23.08 ^a^	0.01	0	7	10	8	21.25 ^c^	0.01
	(3.23%)	(2.42%)	(2.82%)	(1.61%)			(1.61%)	(4.03%)	(4.03%)	(0.40%)			(0.00%)	(2.82%)	(4.03%)	(3.23%)		
41-45 h	24	34	24	22			11	31	38	24			4	42	36	22		
	(9.68%)	(13.71%)	(9.68%)	(8.87%)			(4.44%)	(12.50%)	(15.32%)	(9.68%)			(1.61%)	(16.94%)	(14.52%)	(8.87%)		
46-50 h	14	23	17	12			6	14	28	18			4	27	25	10		
	(5.65%)	(9.27%)	(6.85%)	(4.84%)			(2.42%)	(5.65%)	(11.29%)	(7.26%)			(1.61%)	(10.89%)	(10.08%)	(4.03%)		
>50 h	7	10	19	17			4	8	15	26			7	31	12	3		
	(2.82%)	(4.03%)	(7.66%)	(6.85%)			(1.61%)	(3.23%)	(6.05%)	(10.48%)			(2.82%)	(12.50%)	(4.84%)	(1.21%)		
Night shifts per month																		
0 shift	34	43	40	35	0.56 ^a^	0.91	20	34	58	40	5.66 ^a^	0.13	5	69	49	29	6.27 ^a^	0.10
	(13.71%)	(17.34%)	(16.13%)	(14.11%)			(8.06%)	(13.71%)	(23.39%)	(16.13%)			(2.02%)	(27.82%)	(19.76%)	(11.69%)		
≥1 shifts	19	30	27	20			5	29	33	29			10	38	34	14		
	(7.66%)	(12.10%)	(10.89%)	(8.06%)			(2.02%)	(11.69%)	(13.31%)	(11.69%)			(4.03%)	(15.32%)	(13.71%)	(5.65%)		

a: Pearson’s χ^2^ test, test statistic is χ^2^.

b: Fisher–Freeman–Halton test, test statistic is χ^2^FH.

c: Monte Carlo exact test (100,000 samples) was used because standard Fisher exact enumeration was computationally infeasible, test statistic is χ^2^FH.

### 4.3 Relationship of different variables with occupational fatigue exhaustion/recovery

Table 2 illustrates the positive correlation between chronic fatigue levels and effort and overcommitment scores (*p* < 0.05). Similarly, acute fatigue levels also exhibit a positive correlation with effort and overcommitment scores (*p* < 0.05). In contrast, inter-shift recovery demonstrates a negative correlation with both effort and overcommitment scores (*p* < 0.05). Although effort score and overcommitment score were moderately inter-correlated, auxiliary ordinary least squares regression indicated no problematic multicollinearity (tolerance = 0.61, variance inflation factor = 1.65).

**Table 2:** Correlation analysis of different variables with occupational fatigue exhaustion/recovery.

Items	Degree of chronic fatigue		Degree of acute fatigue		Degree of inter-shift recovery	
	r	*p*	r	*p*	r	*p*
Effort score	0.62	<0.001	0.59	<0.001	−0.51	<0.001
Reward score	−0.1	0.11	−0.02	0.80	0.10	0.13
Overcommitment score	0.53	<0.001	0.56	<0.001	−0.46	<0.001

### 4.4 Regression for levels of occupational fatigue exhaustion/recovery among nursing managers

Regression analysis examined the relationship between participants’ characteristics, work stress and levels of chronic fatigue, acute fatigue, and inter-shift recovery. In this analysis, chronic fatigue, acute fatigue, and inter-shift recovery levels were designated dependent variables, and the participants’ characteristics and work stress variables with statistical significance (*p* < 0.05) in the Chi-Square test or Spearman correlation analysis were classified as independent variables.

Table 3 presents the ordinal regression model for the level of chronic fatigue, *χ*^2^ (4) = 156.20 (*p* < 0.001); no concerning multicollinearity was detected among the variables, with all variance inflation factors (VIFs) ranging from 1.17 to 1.67. Prior to model fitting, the proportional odds assumption was evaluated using the parallel-lines test, *χ^2^* (8) = 8.68 (*p* = 0.37), indicating that the assumption was not violated and the ordinal logistic model was therefore appropriate. The analysis revealed that the effort score (OR = 1.23, 95% CI 1.16–1.31) and overcommitment score (OR = 1.32, 95% CI 1.19–1.46) were significantly associated with chronic fatigue levels. Compared with tertiary hospitals, primary hospitals (OR = 5.11, 95% CI 1.21–21.31) were also associated with higher chronic fatigue. The Cox & Snell R^2^ of the model was 0.47, suggesting that these variables account for approximately 47% of the variation in chronic fatigue levels among nursing managers.

**Table 3:** Associated variables for chronic fatigue levels.

Items	*B*	S. E.	Wald	Sig.	ORs	95% CIs for OR	
						Lower	Upper
Effort score	0.21	0.03	44.28	<0.001	1.23	1.16	1.31
Overcommitment score	0.28	0.06	25.72	0.000	1.32	1.19	1.46
Hospital grade							
Primary	1.63	0.74	4.90	0.027	5.11	1.21	21.78
Tertiary	Reference group						

Table 4 presents the multiple logistic regression model for acute fatigue. Since the overall parallel-lines test was significant *χ*^2^ (26) = 56.88 (*p* < 0.001), we employed multiple logistic regression. The model for acute fatigue, *χ*^2^ (39) = 193.82 (*p* < 0.001); no concerning multicollinearity was detected among the variables, with all variance inflation factors (VIFs) ranging from 1.16 to 1.82. Using high acute fatigue as the reference, higher effort scores were associated with lower odds of low (OR = 0.77, 95% CI 0.66–0.90), mid-low (OR = 0.73, 95% CI 0.64–0.83) and mid-high acute fatigue (OR = 0.84, 95% CI 0.76–0.93), while higher overcommitment scores showed the same downward gradient across these three levels. A ≤ 40 h work-week increased the likelihood than a >50 h of mid-low (OR = 20.00, 95% CI 1.45–275.85) and mid-high fatigue (OR = 15.21, 95% CI 1.46–158.62). Primary hospital and deputy/departmental nurse manager positions, relative to tertiary hospitals and directors of nursing positions, exhibited directionally varied yet consistently protective associations (OR 0.02–0.31, 95% CIs below 1). The Cox & Snell R^2^ was 0.54, suggesting that these variables collectively account for approximately 54% of the variables in acute fatigue levels among nursing managers.

**Table 4:** Associated variables for acute fatigue levels.

Items	*B*	S. E.	Wald	Sig.	ORs	95% CI for OR	
						Lower	Upper
Low							
Effort score	−0.27	0.08	11.29	0.001	0.77	0.66	0.90
Overcommitment score	−0.70	0.14	24.17	<0.001	0.50	0.37	0.66
Hospital grade							
Primary	−23.15	0.00	/	/	8.83E-11	8.83E-11	8.83E-11
Tertiary	Reference group						
Position title							
Deputy nurse managers	−3.76	1.61	5.47	0.02	0.02	0.001	0.54
Departmental nurse managers	−4.09	1.80	5.15	0.02	0.02	<0.001	0.57
Directors of nursing departments	Reference group						
Professional tiltle							
Nurse practitioner	−20.45	0.00	/	/	1.31E-09	1.31E-09	1.31E-09
Professor of Nursing	Reference group						
Mid-low							
Effort score	−0.32	0.07	24.12	<0.001	0.73	0.64	0.83
Overcommitment score	−0.47	0.12	16.07	<0.001	0.63	0.50	0.79
Position title							
Deputy nurse managers	−3.08	1.47	4.37	0.04	0.05	0.003	0.82
Directors of nursing departments	Reference group						
Working hours per week							
≤40 h	3.00	1.34	5.00	0.03	20.00	1.45	275.85
>50 h	Reference group						
Mid-high							
Effort score	−0.18	0.05	11.03	0.001	0.84	0.76	0.93
Overcommitment score	−0.26	0.09	7.79	0.01	0.77	0.65	0.93
Hospital grade							
Primary	−1.17	0.57	4.26	0.04	0.31	0.10	0.94
Tertiary	Reference group						
Working hours per week							
≤40 h	2.72	1.20	5.18	0.02	15.21	1.46	158.62
>50 h	Reference group						
High	Reference group						

Table 5 presents the ordinal regression model for inter-shift recovery, *χ*^2^ (7) = 100.54 (*p* < 0.001); no concerning multicollinearity was detected among the variables, with all variance inflation factors (VIFs) ranging from 1.03 to 1.93. Prior to model fitting, the proportional odds assumption was evaluated using the parallel-lines test, *χ^2^* (14) = 18.15, *p* = 0.20, indicating that the assumption was not violated and the ordinal logistic model was therefore appropriate. The analysis revealed that the effort scores (OR = 0.86, 95% CI 0.81–0.92), overcommitment scores (OR = 0.84, 95% CI 0.76–0.93), and working hours per week were significantly associated with inter-shift recovery levels. The Cox & Snell R^2^ was 0.33, suggesting that these variables collectively account for approximately 33% of the variation in inter-shift recovery levels among nursing managers.

**Table 5:** Associated variables for inter-shift recovery levels.

Items	*B*	S. E.	Wald	Sig.	ORs	95% CI for OR	
						Lower	Upper
Effort score	−0.15	0.03	22.38	<0.001	0.86	0.81	0.92
Overcommitment score	−0.17	0.05	10.48	0.001	0.84	0.76	0.93
Working hours per week							
≤40 h	1.16	0.51	5.20	0.02	3.17	1.17	8.58
41–45 h	0.81	0.36	5.10	0.02	2.24	1.12	4.48
46–50 h	0.84	0.38	4.93	0.03	2.33	1.11	4.90
>50 h	Reference group						

## 5 Discussion

Our findings indicate that western Chinese nursing managers reported significantly elevated occupational fatigue and incomplete inter-shift recovery, specially linked to extrinsic effort and overcommitment. The pattern was likely attributable to severe nursing shortages (11–13, 27–29), and cultural expectations prioritizing collective imperatives over personal recovery. ≥48 h uninterrupted rest is required to restore psychomotor performance (30), but operational demands frequently limit recovery, perpetuating fatigue and hindering inter-shift recovery.

Effort and overcommitment prospectively associated with heightened chronic fatigue level, heightened acute fatigue levels, and diminished inter-shift recovery levels, consistent with prior work that intrinsic effort imbalance induced adverse work experiences (31). The ERR distribution indicated that the managerial role still delivered relative reward. Nevertheless, mid-high to high acute fatigue coexistence signified that even a favorable ERR did not prevent episodic overload. A prospective study found ERI and overcommitment showed no association with occupational burnout (32). Low psychological capital exacerbates fatigue under equivalent workloads (33), whereas Chinese norms of endurance further lower perceived balance thresholds. Role conflict (34) and organizational politics (35), though unmeasured, likely compound acute fatigue among western Chinese nursing managers. The above factors together offer a potential explanatory mechanism for the observed discrepancy.

Across hospital grades, primary hospitals were concurrently linked to heightened chronic fatigue (wide 95% CIs) and to a markedly reduced likelihood of high acute fatigue among western Chinese nursing managers. However, the cross-sectional non-convenience sample and uneven sample distribution across the three hospital grades limit data precision. In China, a tiered healthcare system (primary, secondary, and tertiary) has been established to address regional economic disparities and uneven resource distribution. Bed capacity, patient volume, and case severity increase markedly from primary to tertiary levels (36). Resource constraints leave primary and secondary hospitals with heavier workloads and opaque, unfair management structures (36, 37), resulting in inadequate support for nursing managers.

Position title showed significantly associated with acute fatigue categories among western Chinese nursing managers. Nursing managers often confront complex management situations encompassing personal challenges, patient and family dynamics, organizational demands, and resource constraints (38, 39). Role conflicts and organizational politics may substantially affect their management effectiveness (34, 35).

Weekly working hours were significantly associated with acute fatigue and inter-shift recovery among western Chinese nursing managers, although the wide confidence intervals indicate imprecision, findings consistent with Ma et al. (40). Although the national work standard is 40 h, only 10.08% of nursing managers in our study adhered to a work schedule of 40 h or less per week. Nursing managers remained on-call during ostensible rest (41), eroding recovery opportunities.

National regulatory initiatives that deploy tertiary-grade specialists to primary and secondary facilities have demonstrably attenuated urban–rural disparities in service availability. Concomitantly, robust managerial frameworks, workforce adequacy, and ring-fenced budgets for leadership positions in secondary and primary hospitals remain indispensable for sustaining these gains. Organizational structural support is likely to reduce fatigue-related decision errors and improve care quality. Allowing managers to design self-rostering systems that guarantee at least 24 h of recovery blocks once acute fatigue accumulates (42), alongside flexible duty lines and transferable leave banks, will provide the protected time necessary to interrupt the acute-to-chronic fatigue trajectory. At the individual level, management-training programs should incorporate structured competencies—such as emotional-intelligence (39) and boundary-awareness modules—that may affect occupational fatigue (43, 44). Implementing these combined organizational and personal safeguards may enhance managerial wellbeing, retention, and, ultimately, patient safety.

## 6 Conclusion

Western Chinese nursing managers reported mid-low to mid-high levels of occupational chronic fatigue, mid-high to high levels of acute fatigue, and mid-low to mid-high levels of inter-shift recovery. This study identified low effort and low overcommitment were significantly associated with lightened occupational fatigue and heightened inter-shift recovery. Comprehensive policies allocating institutional resources to western Chinese nursing managers, mandating 24-h recovery periods, and structured competency training programs are needed to reduce occupational fatigue.

## Limitations and further research

Sampling methodology: Using the convenience sampling and online recruitment may result in selection bias and limit the generalizability of the results. Future research would benefit from using stratified random sampling techniques to enhance representativeness.

Cross-sectional design: The study’s cross-sectional nature restricts the ability to establish causal relationships between the variables under investigation. Future studies should consider adopting a prospective study design to facilitate more effective causal analysis.

Subjective measures: The study’s reliance on self-reported measures, without the inclusion of objective evaluation instruments, may introduce recall bias and social desirability effects, potentially affecting the reliability of the findings.

Analytical approach: Treating participants as independent observations across hospitals may overlook intraclass correlation within hospitals and underestimate standard errors. Future studies should adopt multilevel modeling to properly account for hospital grade clustering and yield more accurate inference.

## Data Availability

The original contributions presented in the study are included in the article/Supplementary material (datasets excluded); further inquiries can be directed to the corresponding author.
