# Supplementary material for: Evaluation of occupational fatigue among Chinese nursing managers: a cross-sectional online study
Source: Front Public Health. 2026 Jan 30;14:1752771. doi: 10.3389/fpubh.2026.1752771 (PMC12901362; doi:10.3389/fpubh.2026.1752771)
Supplement: Supplementary file 1 [file Table_1.docx]

**Table S1:** Demographic characteristics of nursing managers

| **Demographic** | **Frequency** | **%** |
| --- | --- | --- |
| Gender |  |  |
| men | 8 | 3.2% |
| women | 240 | 96.8% |
| Age |  |  |
| 20-30 years | 9 | 3.6% |
| 31-40 years | 11 | 4.4% |
| 41-50 years | 132 | 53.2% |
| >50 years | 96 | 38.7% |
| Marital status |  |  |
| unmarried | 3 | 1.2% |
| married | 234 | 94.4% |
| divorced or widowed | 11 | 4.4% |
| Education |  |  |
| three-year college | 17 | 6.9% |
| undergraduate | 208 | 83.9% |
| master's degree or above | 23 | 9.3% |
| Hospital location |  |  |
| eastern part | 11 | 4.4% |
| central part | 11 | 4.4% |
| western part | 226 | 91.1% |
| Hospital grade |  |  |
| primary | 9 | 3.6% |
| secondary | 45 | 18.2% |
| tertiary | 194 | 78.2% |
| Years of experience |  |  |
| 6-10 years | 32 | 12.9% |
| 11-20 years | 110 | 44.4% |
| >20 years | 106 | 42.7% |
| Position title |  |  |
| deputy nurse managers | 61 | 24.6% |
| nurse managers | 142 | 57.3% |
| departmental nurse managers | 26 | 10.5% |
| directors of nursing departments | 19 | 7.7% |
| Professional title |  |  |
| nurse practitioner | 14 | 5.7% |
| nurse-in-charge | 139 | 56.1% |
| associate professor of nursing | 85 | 34.3% |
| professor of nursing | 10 | 4.0% |
| Department |  |  |
| outpatient | 26 | 10.5% |
| emergency | 4 | 1.6% |
| operating room | 10 | 4.0% |
| intensive care unit | 5 | 2.0% |
| internal medicine | 17 | 6.9% |
| surgery | 12 | 4.8% |
| gynecology | 23 | 9.3% |
| obstetrics | 91 | 36.7% |
| pediatrics | 31 | 12.5% |
| non-clinical departments | 29 | 11.7% |
| Working hours per week |  |  |
| ≤40h | 25 | 10.1% |
| 41-45h | 104 | 41.9% |
| 46-50h | 66 | 26.6% |
| >50h | 53 | 21.4% |
| Night shifts per month |  |  |
| 0 shift | 152 | 61.3% |
| 1-4 shifts | 83 | 33.5% |
| ≥5 shifts | 13 | 5.2% |
