# Supplementary material for: Evaluation of occupational fatigue among Chinese nursing managers: a cross-sectional online study
Source: Front Public Health. 2026 Jan 30;14:1752771. doi: 10.3389/fpubh.2026.1752771 (PMC12901362; doi:10.3389/fpubh.2026.1752771)
Supplement: Supplementary file 2 [file Table_2.docx]

**Table S2:** Frequency and percentage of variables on OFER

| **Variables** |  | **Groups** | **N** | **(%)** |
| --- | --- | --- | --- | --- |
| OFER |  |  |  |  |
|  | Chronic Fatigue | low | 53 | (21.4%) |
|  |  | mid-low | 73 | (29.4%) |
|  |  | mid-high | 67 | (27.0%) |
|  |  | high | 55 | (22.2%) |
|  | Acute Fatigue | low | 25 | (10.1%) |
|  |  | mid-low | 63 | (25.4%) |
|  |  | mid-high | 91 | (36.7%) |
|  |  | high | 69 | (27.8%) |
|  | Inter-shift Recovery | low | 15 | (6.1%) |
|  |  | mid-low | 107 | (43.2%) |
|  |  | mid-high | 83 | (33.5%) |
|  |  | high | 43 | (17.3%) |
