# Supplementary material for: Evaluation of occupational fatigue among Chinese nursing managers: a cross-sectional online study
Source: Front Public Health. 2026 Jan 30;14:1752771. doi: 10.3389/fpubh.2026.1752771 (PMC12901362; doi:10.3389/fpubh.2026.1752771)
Supplement: Supplementary file 3 [file Table_3.docx]

**Table S3:** Analysis of variables scores and ERR (Frequency and Percentage) on ERI

| **Variables** |  | **Groups** | **Median** | **(Q1 - Q3)** |  |  | **N** | **(%)** |
| --- | --- | --- | --- | --- | --- | --- | --- | --- |
| ERI |  |  |  |  |  |  |  |  |
|  | Effort score | / | 19 | (16 - 23) |  |  | / |  |
|  | Reward score | / | 39 | (37 - 41) |  |  | / |  |
|  | Overcommitment score | / | 17 | (16 - 19) |  |  | / |  |
|  | ERR | ≤1 | 0.78 | (0.61 - 0.89) |  |  | 161 | (64.9%) |
|  |  | ＞1 | 1.19 | (1.07 - 1.32) |  |  | 87 | (35.1%) |
